# RNF114, a RING E3 ligase that reads and extends the hybrid ADP-ribose-ubiquitin signal

**DOI:** 10.1038/s44318-025-00576-0

**Published:** 2025-10-02

**Authors:** Adam J Middleton, Catherine L Day

**Affiliations:** Department of Biochemistry, University of Otago, Dunedin, 9054 New Zealand

**Keywords:** Post-translational Modifications & Proteolysis, Structural Biology

## Abstract

Several recent studies report the characterization of a novel domain recognizing both ADPr and ubiquitin, enabling ubiquitin ligases to catalyze further modification of this posttranslational mark.

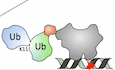

## Teaser

Post-translational modifications (PTMs) expand protein function and serve as key regulators of virtually all cellular processes. Several recent studies show that the E3 ligase RNF114 recognises a hybrid ADP-ribose-ubiquitin modification on substrate proteins and uses it as a platform to assemble Lys11-linked ubiquitin chains, placing RNF114 at the crossroads of DNA repair processes that help cells safeguard their genome and respond to stress.

The addition of ADP-ribose (ADPr) to proteins –ADP-ribosylation– serves as a crucial stress signal in eukaryotes (Suskiewicz et al, 2023). At sites of DNA damage, enzymes of the poly(ADP-ribose) polymerase (PARP) and Tankyrase families, including PARP1, PARP2, TNKS1, and TNKS2, catalyze the covalent addition of single molecules of ADPr or chains, known as poly(ADP-ribose) (PAR), to proteins (and nucleic acids). The addition of ADPr and PAR is important because proteins involved in DNA repair often contain ADPr- or PAR-binding domains that target them to sites requiring repair. While the addition of ADPr and PAR is critical for normal cellular health, it creates a therapeutic vulnerability in cancer patients with BRCA1/2 mutations and homologous-recombination deficiencies, because DNA repair broadly relies on PARP-dependent processes. As a result, PARP inhibitors that block the addition of ADPr and PAR chains, and that trap PARP enzymes on DNA, are synthetic-lethal with the primary defect and therefore commonly used for treatment of BRCA-deficient cancers.

The success of PARP inhibitors also highlights the importance of resolving the ADPr signal once DNA repair is complete. While hydrolases that remove ADPr and PAR have been identified, quenching of the ADPr signal also relies on degradation of the modified protein. Most notably, the E3 ligase RNF146 binds proteins modified with PAR chains via its WWE domain, which promotes ubiquitin transfer by the RING domain, leading to the ubiquitylation and subsequent degradation of substrate proteins (DaRosa et al, 2015). More recently, several studies suggested that another RING E3 ligase, referred to as RNF114, promotes the ubiquitylation of ADPr-modified proteins at sites of DNA damage (Li et al, 2023; Longarini et al, 2023; Perrard and Smith, 2023). The importance of RNF114 was highlighted by the discovery that inhibition of RNF114 via nimbolide (Li et al, 2023), like inhibition of PARP enzymes with PARP inhibitors, kills cells that have BRCA1/2 mutations. Now, a series of new reports have used elegant biochemical approaches to help advance our molecular understanding of RNF114 function.

**Fig. 1:**
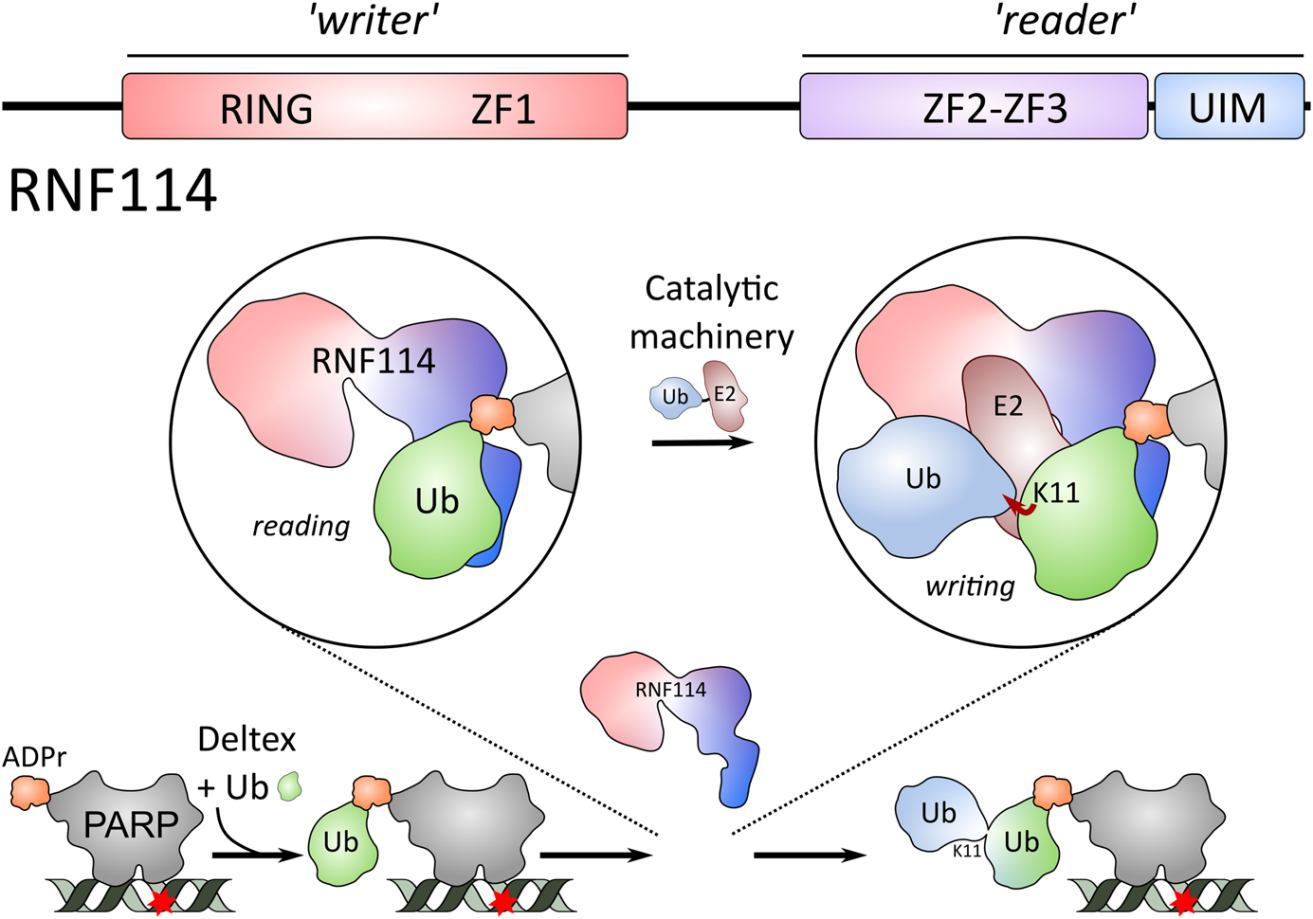
RNF114 a reader and writer of the ubiquitin code. RNF114 is a multidomain protein, with the N-terminal ‘writer’ domains involved in promoting the addition of ubiquitin to substrate proteins, while the C-terminal ‘reader’ domains bind the ADPr-Ub signal. This means that RNF114 is only targeted to substrates—such as PARP1 as shown here—following their prior modification with the ADPr-Ub hybrid signal. Current studies focus on the role of RNF114 in DNA repair because PARP1, which adds ADPr, has a well-established role in detecting breaks (red star) in DNA.

At the basis of this focus on RNF114 is the discovery that the Deltex (DTX) family of E3 enzymes link ubiquitin to ADPr on substrates proteins, resulting in the formation of the hybrid ADPr-ubiquitin (ADPr-Ub) modification (Zhu et al, 2022). Several new studies, including one in this issue of *The EMBO Journal* (Kloet et al, 2025; Kolvenbach et al, 2025; Lacoursiere et al, 2025; Perrard et al, 2025), now show that while RNF114 can interact with both mono-ADPr and ubiquitin, it binds specifically and tightly to the hybrid ADPr-Ub modification. Not only does RNF114 bind ADPr-Ub, but it also further modifies it, resulting in the formation of ADPr-Ub-Ub, with a Lys11 link connecting the two ubiquitin molecules (Lacoursiere et al, 2025). Together, these papers establish RNF114 as both *reader* and *writer* of PTMs in DNA repair pathways (Fig. 1).

RNF114 belongs to a small family of RING E3 ligases that includes RNF125, RNF138, and RNF166 (Giannini et al, 2008). All four proteins have an N-terminal RING domain followed by three zinc fingers (ZFs) and a C-terminal ubiquitin interaction motif (UIM) (Fig. 1). The RING domain together with the first ZF promotes ubiquitin transfer and efficiently assembles degradative ubiquitin chains (Middleton et al, 2023). The function of the C-terminal domains has been less clear, although it was generally assumed that the UIM may bind ubiquitin or ubiquitin chains to target the E3 ligase activity to certain substrates. However, the new work shows that the UIM works together with ZF2 and ZF3 (ZF2/3) to bind substrates as ADPr binds in a pocket formed by ZF2/3, while ubiquitin binds to the UIM (Lacoursiere et al, 2025).

Central to the recent discoveries was the development of several powerful reagents that will likely lead to the elucidation of additional machinery involved in the writing and decoding of ADPr-Ub signals. Perhaps most importantly, Kloet et al, (2025) have developed a method that enables the synthesis of a non-hydrolyzable ADPr-Ub analog. Using the ADPr-Ub analog in an unbiased proteomics discovery approach resulted in the enrichment of RNF114, RNF138, and RNF166 in the suite of bound proteins. Taking a complementary approach, Kolvenbach et al, (2025) started with GFP-tagged RNF114 and looked for binding partners that relied on the integrity of the ZF2/3 domains. This led to the identification of several DNA repair enzymes and the subsequent development of an optimized mass-spectrometry approach that allows sites in proteins modified with ADPr-Ub to be identified. Meanwhile, Lacoursiere et al, (2025) developed a click-chemistry chemoenzymatic-based approach for the synthesis of a fluorescent ADPr-Ub probe, which can be used to study ADPr-Ub binding and activity.

Using these tools and high-confidence structural modeling via AlphaFold 3, the critical role of ZF2/3 and the UIM of RNF114 in recognition of ADPr-Ub has been revealed, along with the ability of RNF114 to add ubiquitin to ADPr-Ub, favoring formation of Lys11-linked diubiquitin. The model of the assembled catalytic complex, containing RNF114 bound to ubiquitin and ADPr as well as components of the ubiquitin cascade, revealed that Lys11 of the incoming ubiquitin molecule was directed towards the active site of the activated complex (Fig. 1). Supported by site-directed mutagenesis, this model accounts for how RNF114 interacts with ADPr-Ub and explains how RNF114 can specify the synthesis of Lys11-linked ubiquitin chains (Lacoursiere et al, 2025). Furthermore, Kloet et al show that disrupting either the UIM or ZF2/3 in RNF114 abolishes interaction with ADPr-Ub, emphasizing that the interaction is highly dependent on binding both components of the hybrid ADPr-Ub moiety (Kloet et al, 2025). While the focus has been on RNF114, RNF166 appears to have comparable activity. In contrast, another member of the family, RNF125, has a divergent ZF2/3 binding pocket and appears not to bind ADPr-Ub. The new tools, as well as further cellular studies, will undoubtedly help untangle the role played by each member of this emerging E3 ligase family.

Although the recent publications significantly advance our understanding of RNF114 function, there remains much to discover. For example, what is the preferred residue of ADPr-Ub modification that recruits RNF114? ADPr-Ub attached to glutamate and aspartate residues is suggested as the primary target of RNF114 by Lacoursiere et al, (2025), on the other hand, Perrard et al, (2025) and Kolvenbach et al, (2025) propose that RNF114 primarily binds to ADPr-Ub that is attached to serine. While there may be specificity, the models of RNF114 bound to ADPr-Ub indicated that the sidechain may not be itself recognized, and it seems likely that RNF114 will “read” ADPr-Ub on various sidechains. The nature of the key substrate proteins that are modified with ADPr-Ub also needs to be revealed. Mass-spectrometry approaches have identified an array of proteins that interact with RNF114 in an ADPr-Ub-dependent manner (Kolvenbach et al, 2025), but validation and further analysis of these potential substrates is now required. Once RNF114 is recruited, what is the fate of substrate proteins? Early studies suggested that RNF114 ubiquitylates proteins and targets them for degradation, but Perrard et al, (2025) show that when RNF114 and RNF166 bind ADPr-Ub, this stabilizes the bound proteins by preventing the formation of PAR chains and interaction with degradation-inducing RNF146 (Perrard et al, 2025). There is strong evidence indicating RNF114 catalyzes only Lys11-linkages between ubiquitin moieties (Kloet et al, 2025; Lacoursiere et al, 2025), and while Lys11-linked chains are normally a proteasomal degradation signal, it requires further investigation whether longer chains are actually formed on substrates inside cells. New tools, including those described in the recent studies, will undoubtedly allow further insight into RNF114 function in the next few years. It also seems likely that additional readers of ADPr-Ub will be identified, and understanding how specificity is achieved will be of considerable interest.

There is still much to learn about the interplay between PTMs, but the importance of RNF114 as a reader of ADPr-Ub and a writer of Lys11-linked chains is without question. Future studies will be required to more clearly define the cellular function of RNF114, and they may also pave the way for the development of new therapeutic approaches. RNF114 is an attractive target because its deletion, or its inhibition by nimbolide, traps PARP1 on DNA and kills BRCA1/2-deficient tumor cells that are resistant to PARPi (Li et al, 2023; Longarini et al, 2023). To date, efforts to target RNF114 have focused on the development of nimbolide analogs, with nimbolide-based compounds used to both inhibit RNF114 activity and to build proteolysis-targeting chimeric molecules (PROTACS) (Luo et al, 2021; Spradlin et al, 2019). However, the recent discoveries suggest that ADPr analogs may have potential as RNF114 inhibitors or as novel recruiters, with applications in targeted protein degradation. Together, the recent articles position RNF114 as a central decoder of ADPr-Ub signals that have significant implications for biology and potential applications for the treatment of disease.

## References

[CR1] DaRosa PA, Wang Z, Jiang X, Pruneda JN, Cong F, Klevit RE, Xu W (2015) Allosteric activation of the RNF146 ubiquitin ligase by a poly(ADP-ribosyl)ation signal. Nature 517:223–226 25327252 10.1038/nature13826 PMC4289021

[CR2] Giannini AL, Gao Y, Bijlmakers MJ (2008) T-cell regulator RNF125/TRAC-1 belongs to a novel family of ubiquitin ligases with zinc fingers and a ubiquitin-binding domain. Biochem J 410:101–111 17990982 10.1042/BJ20070995 PMC2733222

[CR3] Kloet MS, Chatrin C, Mukhopadhyay R, van Tol BDM, Smith R, Rotman SA, Tjokrodirijo RTN, Zhu K, Gorelik A, Maginn L et al (2025) Identification of RNF114 as ADPr-Ub reader through non-hydrolysable ubiquitinated ADP-ribose. Nat Commun 16:6319 40634336 10.1038/s41467-025-61111-7 PMC12241653

[CR4] Kolvenbach A, Palumbieri MD, Colby T, Nadarajan D, Bode R, Matic I (2025) Serine ADPr on histones and PARP1 is a cellular target of ester-linked ubiquitylation. Nat Chem Biol. 10.1038/s41589-025-01974-5 10.1038/s41589-025-01974-5 PMC12568645 40634527

[CR5] Lacoursiere RE, Upadhyaya K, Sidhu JK, Bejan DS, Siordia IR, Cohen MS, Pruneda JN (2025) RNF114 and RNF166 exemplify reader-writer E3 ligases that extend K11 polyubiquitin onto sites of MARUbylation. EMBOJ 44. 10.1038/s44318-025-00577-z 10.1038/s44318-025-00577-z PMC12583694 41039157

[CR6] Li P, Zhen Y, Kim C, Liu Z, Hao J, Deng H, Deng H, Zhou M, Wang XD, Qin T et al (2023) Nimbolide targets RNF114 to induce the trapping of PARP1 and synthetic lethality in BRCA-mutated cancer. Sci Adv 9:eadg7752 37878693 10.1126/sciadv.adg7752 PMC10599614

[CR7] Longarini EJ, Dauben H, Locatelli C, Wondisford AR, Smith R, Muench C, Kolvenbach A, Lynskey ML, Pope A, Bonfiglio JJ et al (2023) Modular antibodies reveal DNA damage-induced mono-ADP-ribosylation as a second wave of PARP1 signaling. Mol Cell 83:1743–1760.e1711 37116497 10.1016/j.molcel.2023.03.027 PMC10205078

[CR8] Luo M, Spradlin JN, Boike L, Tong B, Brittain SM, McKenna JM, Tallarico JA, Schirle M, Maimone TJ, Nomura DK (2021) Chemoproteomics-enabled discovery of covalent RNF114-based degraders that mimic natural product function. Cell Chem Biol 28:559–566.e515 33513350 10.1016/j.chembiol.2021.01.005 PMC8052289

[CR9] Middleton AJ, Barzak FM, Fokkens TJ, Nguyen K, Day CL (2023) Zinc finger 1 of the RING E3 ligase, RNF125, interacts with the E2 to enhance ubiquitylation. Structure 31:1208–1219.e1205 37541247 10.1016/j.str.2023.07.007

[CR10] Perrard J, Gao K, Ring K, Smith S (2025) Deltex and RING-UIM E3 ligases cooperate to create a ubiquitin-ADP-ribose hybrid mark on tankyrase, promoting its stabilization. Sci Adv 11:eadx7172 40901936 10.1126/sciadv.adx7172 PMC12407064

[CR11] Perrard J, Smith S (2023) Multiple E3 ligases control tankyrase stability and function. Nat Commun 14:7208 37938264 10.1038/s41467-023-42939-3 PMC10632493

[CR12] Spradlin JN, Hu X, Ward CC, Brittain SM, Jones MD, Ou L, To M, Proudfoot A, Ornelas E, Woldegiorgis M et al (2019) Harnessing the anti-cancer natural product nimbolide for targeted protein degradation. Nat Chem Biol 15:747–755 31209351 10.1038/s41589-019-0304-8 PMC6592714

[CR13] Suskiewicz MJ, Prokhorova E, Rack JGM, Ahel I (2023) ADP-ribosylation from molecular mechanisms to therapeutic implications. Cell 186:4475–4495 37832523 10.1016/j.cell.2023.08.030 PMC10789625

[CR14] Zhu K, Suskiewicz MJ, Hlousek-Kasun A, Meudal H, Mikoc A, Aucagne V, Ahel D, Ahel I (2022) DELTEX E3 ligases ubiquitylate ADP-ribosyl modification on protein substrates. Sci Adv 8:eadd4253 36197986 10.1126/sciadv.add4253 PMC7615817

